# Cohort Profile Update: The 1993 Pelotas (Brazil) Birth Cohort follow-up at 22 years

**DOI:** 10.1093/ije/dyx249

**Published:** 2017-12-11

**Authors:** Helen Gonçalves, Fernando C Wehrmeister, Maria C F Assunção, Luciana Tovo-Rodrigues, Isabel O de Oliveira, Joseph Murray, Luciana Anselmi, Fernando C Barros, Cesar G Victora, Ana M B Menezes

**Affiliations:** 1 Postgraduate Program in Epidemiology, Universidade Federal de Pelotas, Pelotas, Brazil; 2 Postgraduate Program in Health and Behavior, Universidade Católica de Pelotas, Pelotas, Brazil

## The original cohort

All births occurring in Pelotas (*N* = 5265), from 1 January to 31 December 1993, were enrolled and 5249 agreed to take part in the longitudinal study. In the original cohort profile [http://ije.oxfordjournals.org/content/37/4/704.short]^1^ we described how all live-born children in 1993 in the city of Pelotas, Brazil, were followed up until 11 years of age, and later we updated in a new profile^2^ reporting on the ages of 15 and 18 years.

## What is the reason for the new focus (or new data collection)? 

The preceding visit to the cohort took place in 2011, when cohort participants were 18 years old. Giving sequence to the follow-up, the new visit was the first to study the young adulthood period, at the age of 22 years. The study was expanded to include more detailed assessments of human capital, cognitive function, mental health, cardiovascular assessment, reproductive history and biochemical and genetic characteristics.

Special attention was given to the study of precursors and risk factors for non-communicable diseases (NCD) which are now the leading cause of death in Brazil^3^ and worldwide.^4^ The study of inequalities in health has been a key objective of the cohort since its inception, and the new visit allows us to measure disparities among young adults. Most variables assessed in 2015 had also been evaluated in previous follow-ups,^2^ and will thus allow the study of trajectories in health status and exposure to risk factors over time. This profile updates earlier descriptions of the 1993 cohort.^2^

## What will be the new areas of research? 

The main focus of the visit at 22 years was described above. New areas of research, that were not part of the earlier visits, include:

- mental health (e.g. anhedonia, well-being, post-traumatic stress);
- information on sleep quality and sleep problems;
- intensity of effect of adverse or stressful life events (e.g. assault, domestic violence, parental death, disruption of romantic relationships);
- use of social media (what and when);
- hookah use (flavoured tobacco);
- restless legs syndrome;
- asthma control;
- headache (type, intensity, symptoms);
- violence suffered (inside and outside family) and perpetrated;
- health plan (type and covering what);
- consumption of alcohol and smoking during pregnancy (woman and partner);
- diffusing capacity of the lungs for carbon monoxide (DLCO);
- pulse wave velocity (PWV).

In addition, blood samples were taken and stored in ultra-low temperature freezers for future biochemical, genetic and epigenetic analyses.

Furthermore, during the follow-up at 22 years a sub-study was begun which marks the continuity of intergenerational health research, the first follow-up of the so-called second generation of the 1993 cohort (93Cohort-II). It will expand the capacity of researchers to explore and identify mechanisms associated with parental health which affect the health of children, as well as their relationships with socioeconomic and environmental changes, in two or three generations (mothers of cohort members, cohort members and children of cohort members) and in other cohorts, such as ALSPAC (Children’s Children of the 90s)^5^ and Birth to Twenty (3G children).^6^ Of the total 1650 children identified as offspring, 1213 were evaluated.

With support from the Brazilian government, the 1993 cohort has coordinated data collection with two other cohorts, in São Luís (Maranhão State) and Ribeirão Preto (São Paulo State),^7^^,^^8^ at a similar age to the 1993 cohort members, denominated **R**ibeirão Preto, **P**elotas e **S**ão Luís Consortium of Brazilian Cohorts [RPS Consortium of Brazilian Cohorts]; that is, three birth cohort studies collected new data in a similar period for future comparisons. The main objective of this collaboration is to investigate the early determinants of health and nutritional status in childhood, adolescence and adulthood across geographically, culturally and economically distinct regions of the country. Moreover, data from this cohort are part of the Consortium of Health-Oriented Research in Transitioning Societies (COHORTS),^9^ which aims in particular to strengthen collaboration between five of the largest and longest birth cohort studies in low- and middle-income countries.

## Who is in the cohort? 

**Figure 1:**
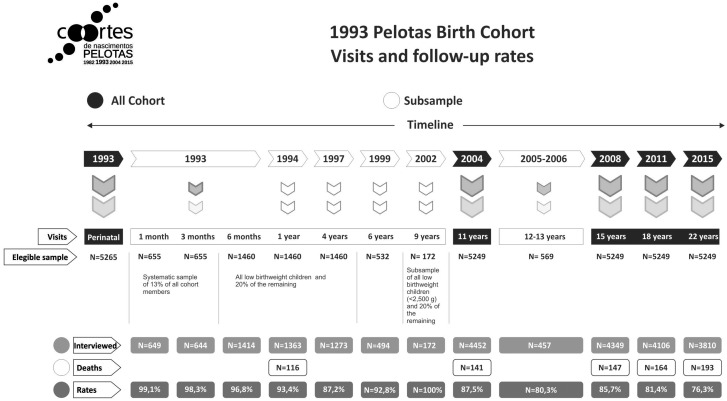
Description of the 1993 Pelotas Birth Cohort. Visits and follow-up rates.

The original cohort included all live-born children delivered in one of the city’s hospitals in 1993, whose mothers lived in the urban area of Pelotas as delimited in 1982, when the first cohort was formed.^10^ Over 99% of all births occur in a hospital. Follow-up visits are shown in Figure 1.

Shortly after the end of the 18-year follow-up, personal identification and contact data began to be updated through searches in phone directories, social networks and governmental online registers. When a cohort member could not be located, we contacted known relatives or friends using information provided by the subject at the previous visit. The next step was for a member of the study team to visit the last known address and to speak with neighbours to identify the new place of residence. During 2015, announcements were made in the local media (TV, radio and newspaper) and social networks were used to encourage participants to contact the study headquarters and receive information on the new visit.

The 22-year follow-up visit started in October 2015 and finished in July 2016. Of the original cohort of 5249 subjects, 4933 were found. All located members were invited to attend the cohort clinic and participate in the follow-up visit; 3810 were interviewed, 175 (3.5%) refused to participate and 1071 (21.7%) were considered losses. Those who completed the interviews, added to those known to have died (*N* = 193), represent a retention rate of 76.3% (Figure 1).

The average age of the interviewees was 22.6 years (range 21.9–23.5 years). Table 1 presents follow-up rates at age 22 according to selected health and sociodemographic variables measured at birth. At least 69% of subjects in each subgroup were located. Losses to follow-up were higher among males and at the extremes of the income distribution.

**Table 1:** Follow-up rates at 22 years according to baseline characteristics 1993 Birth Cohort, Pelotas (Brazil)

Variable	Original sample (1993)	% interviewed in 2015	P*	% located (interviewed in 2015 + known to have died)	P*
Sex	5248		< 0.001		< 0.001
Male	2603	68.5		72.6	
Female	2645	76.6		79.9	
Birthweight (g)	5232		0.014		0.406
<2500	510	66.9		79.2	
2500–2999	1409	72.5		75.7	
3000–3499	2030	73.6		75.9	
≥3500	1283	74.0		76.0	
Gestational age (weeks)	5171		0.002		0.205
<37	589	67.1		74.1	
≥37	4582	73.3		76.5	
Household income^a^	5249		< 0.001		0.040
≤1	967	68.5		75.1	
1.1 to 3.0	2260	71.6		74.8	
3.1 to 6.0	1204	77.3		79.6	
6.1 to 10	433	73.0		75.7	
>10	385	73.5		74.6	
Maternal schooling (years)	5246		< 0.001		0.068
0	134	62.31		69.3	
1 to 4	1338	69.36		74.6	
5 to 8	2424	73.60		77.0	
≥9	1350	74.96		77.3	
Total	5249	72.6	< 0.001	76.3	< 0.001

^a^1 = minimum wage.

*Chi square test.

## What has been measured? 

### Interviews

The questionnaires covered variables related to health, schooling, family composition, behaviour and lifestyle. Table 2 shows the main categories assessed and which staff collected each type of data.

**Table 2:** Main categories of variables collected in the most recent follow-up visits, Pelotas 1993 Birth Cohort Study

Instrument	Variables
Interview	School achievement; employment and salary; family composition; family income; bone fractures; self-perceived health; self-perception of oral health; body image; injuries; nicotine consumption including cigarette smoking; alcohol intake; physical activity; morbidity history and hospitalizations; use of health services and of medicines; offspring (date of birth, birthweight, breastfeeding duration, delivery, number of children); marital status; assessment of relationship with partner; head injury; restless legs syndrome; sleep problems and drowsiness; parental morbidity and mortality; body pain; headaches; use of social media; wheezing and asthma; health insurance; common mental disorders (Self-Reporting Questionnaire, SRQ-20)
Confidential	Stressful life events; violence (suffered and perpetrated); illicit drug use; use of contraceptive methods; history of abortions; number of sexual partners
Self-reported	Food frequency; facial expression recognition task (Brazilian version)
Psychological interview (test)	Mini International Neuropsychiatric Interview (M.I.N.I.): major depressive episode, suicide attempt, bipolar, social phobia, generalized anxiety disorder, attention-deficit/hyperactivity disorder, post-traumatic stress, antisocial personality; Digit Span (subtest WAIS-III); DSM-5 Self-Rated Level 1 Cross-Cutting Symptom; Snaith-Hamilton Pleasure Scale; Center for Epidemiologic Studies Depression Scale (CESD-R); Well-being (Warwick-Edinburgh Mental Well-being Scale, WEMBWS)
Physical examinations	Weight, height, BMI, sitting height; waist circumference; whole-body three-dimensional photonic scanning (3D photonic scanner); blood pressure; blood samples; aortic pulse wave velocity, dynamometry, accelerometry; spirometry; diffusing capacity of the lungs for carbon monoxide; dual-energy X-ray absorptiometry (DXA Lunar Prodigy): fat mass, free fat mass, bone density and content; air displacement plethysmography (BodPod): fat mass, lean mass

Mental health questionnaires were applied by psychologists. Eight types of mental disorders were evaluated using the Mini International Neuropsychiatric Interview (M.I.N.I.): major depressive episode, social anxiety disorder, attention-deficit/hyperactivity disorder, bipolar disorder, post-traumatic stress disorder, antisocial personality disorder, generalized anxiety disorder and lifetime suicidal attempt. In addition, the following tests were used.

- Anhedonia was measured by the short version of Snaith-Hamilton Pleasure Scale (SHAPS).^11^
- The Span of Digits test, a subtest of the Wechsler’s Intelligence Scales for Adults (WAIS-III),^12^^,^^13^ was used to evaluate working memory.
- Well-being was assessed using the Warwick-Edinburgh Mental Well-being Scale (WEMBWS) which measures the positive attributes of well-being, covering hedonic and eudaimonic perspectives.^14^^,^^15^
- The Facial Expression Recognition Brazilian Task (FERBT)^16^ was applied to evaluate the ability to recognize facial expressions of emotion, using computer-displayed photographs of Brazilian actors portraying facial expressions of fear, sadness, happiness, repugnance, surprise and anger.

During the follow-up visits, we carried out a validation study of DSM-5 Self-Rated Level 1 Cross-Cutting Symptom measure,^17^ commonly used in clinical practice. The test was applied to all young adults interviewed; its validation may encourage its application in primary care settings.

Two instruments were used to evaluate sleep problems: the Brazilian Portuguese version of the Pittsburgh Sleep Quality Index (PSQI),^18^^,^^19^ a scale that evaluates quality and sleep disorders in the previous month, and the Epworth Sleepiness Scale,^20^ in its Brazilian version,^21^ to measure recent daytime.

### Physical examinations and biomarkers

Data were collected for the evaluation of body composition using 3D Photonic scanner (3D Whole Body Scanner, USA), dual-energy X-ray absorptiometry (DXA Lunar Prodigy, USA), air-displacement plethysmography (BodPod^®^ Gold Standard, USA) and traditional anthropometry (weight, sitting height and waist circumference). Other measurements included: blood pressure (model HEM-705CPINT, OMRON, Beijing; margin of error: 1 mmHg), accelerometry (ActiGraph, wGT3X-BT, wGT3X and ActiSlee models, USA) and spirometry (ndd Easyone, Switzerland). All of these measurements had also been obtained in the preceding visit at the age of 18 years.^2^

The new measurements introduced at the 22-year follow-up visit were: aortic pulse wave velocity (SphygmoCor, Atcor Medical, V9.0, Australia), grip strength using dynamometry (Jamar Plus^®^, USA) and diffusing capacity of the lungs for carbon monoxide (EasyOne Pro^™^, UK).

Blood samples were drawn by venepuncture using vacutainer tubes. All samples were processed in the laboratory, stored at ultra-low temperature freezers in the same place and registered in a central biorepository. Genomic DNA was extracted by the salting-out method from whole blood samples and aliquots were also stored at −80°C. Several biomarkers of cardiometabolic risk [glucose, cholesterol, triglycerides, high-density lipoprotein (HDL) cholesterol, low-density lipoprotein (LDL) cholesterol, uric acid], renal function (urea, creatinine), liver enzymes [gamma-glutamyl transferase (GGT), alanine aminotransferase (ALT), aspartate aminotransferase (AST)], hormones (insulin and oxytocin) and inflammation markers [ultrasensitive C-reactive protein (CRP), homocysteine] will be assessed using serum samples. In addition to these, fibrinogen levels (clotting factor) were measured in citrate plasma samples.

The average time required for the entire data collection was 4 h, during which time refreshments are provided. The questionnaires used in the present and in previous visits are available at [http://www.epidemio-ufpel.org.br/site/content/coorte_1993-en/questionnaires.php] (in Portuguese).

### Ethics

Ethical approval was obtained from the Medical School Ethics Committee of the Federal University of Pelotas and fully informed consent was provided by cohort members.

## What has been found? Key findings and publications

The prevalence of some of the key measures obtained at the 22-year follow-up are presented for males and females in Table 3. Other indicators are still being analysed and will be reported in future articles.

**Table 3:** Selected characteristics of the study sample at the 22-year visit: mental health, human capital, body composition and precursors of complex chronic diseases variables (*N* = 3810)

Variable	*N*	Men	Women	*P*
Mental health				
Common mental disorders, % (95% CI)	3784	19.6 (17.7; 21.4)	24.1 (22.2; 26.0)	0.001
Major depressive episode (with impairment), % (95% CI)	3781	1.4 (0.8; 1.9)	4.2 (3.3; 5,0)	< 0.001
Attention-deficit/hyperactivity disorder (with impairment), % (95% CI)	3780	4.1 (3.2; 5.0)	4.8 (3.9; 5.7)	0.282
Warwick-Edinburgh Mental Well-being Scale,^a^ mean (95% CI)	3515	53.1 (52.6; 53.5)	50.0 (49.5; 50.4)	< 0.001*
Human capital				
Achieved schooling (complete years), mean (95% CI)	3805	9.3 (9.2; 9.5)	10.1 (10.0;10.2)	< 0.001*
Employed, % (95% CI)	3810	73.5 (71.4; 75.5)	53.9 (51.7; 56.0)	< 0.001
Body composition/anthropometry				
Fat mass percentage (BodPod), mean (95% CI)	3559	20.9 (20.4; 21.3)	35.8 (35.4; 36.2)	< 0.001*
Body mass index (kg/m^2^), mean (95% CI)	3559	25.0 (24.8; 25.2)	25.5 (25.2; 25.7)	0.008*
Prevalence of overweight (BMI 25–29.9 kg/m^2^), % (95% CI)	3559	29.6 (27.5; 31.81)	24.6 (22.6; 26.5)	< 0.001
Prevalence of obesity (BMI ≥ 30 kg/m^2^), % (95% CI)	3559	13.5 (11.8; 15.1)	18.7 (16.9; 20.4)	< 0.001
Precursors of chronic disease				
Prevalence of tobacco smoking, % (95% CI)	3805	20.3 (18.4; 22.1)	13.7 (12.2; 15.2)	< 0.001
Physical inactivity^a^ (< 150 min/week), % (95% CI)	3797	25.6 (23.6; 27.6)	42.7 (40.5; 44.8)	< 0.001
Poor sleep quality (PSQI scale ≥ 6), % (95% CI)	3796	40.4 (38.1; 42.7)	47.8 (45.6; 49.9)	< 0.001
Systolic blood pressure^b^ (mm Hg), mean (95% CI)	3589	131.6 (131.0; 132.2)	116.9 (116.4; 117.4)	< 0.001*
Diastolic blood pressure^b^ (mmHg), mean (95% CI)	3591	73.8 (73.4; 74.2)	72.4 (72.0; 72.8)	< 0.001*
Forced expiratory volume in first second (L), mean (95% CI)	3509	4.12 (4.09; 4.16)	3.00 (2.98; 3.02)	< 0.001*
Carbon monoxide diffusing capacity (ml/min/mm Hg), mean (95% CI)	2741	32.9 (32.6; 33.2)	21.7 (21.5; 21.9)	< 0.001*

PSQI, Pittsburgh Sleep Quality Index; CI, confidence interval.

^a^Total physical activity = leisure + commuting.

^b^Mean between two valid measures.

*P*-values by chi square test, except *P-values by t-test.

### Mental health and human capital

Women, compared with men, had a higher prevalence of common mental disorders (using Self-Reporting Questionnaire SRQ-20),^22^ major depressive episodes and attention-deficit/hyperactivity disorder (Table 3). A greater proportion of men had high well-being scores compared with women. There was no difference in numbers of full years of schooling between men and women at age 22, but more men (74%) than women (54%) were employed. 

### Body composition/anthropometry

The percentage of body fat mass was higher in females than males, as well as the prevalence of obesity [body mass index (BMI) > 30 kg/m^2^]. However, the reverse was observed for overweight: about 30% of the young men and 25% of the women had BMI > 25 kg/m^2^ (Table 3).

### Precursors of chronic disease 

As shown in Table 3, men reported smoking (tobacco) more frequently than women (20% and 14%, respectively). According to the WHO recommendation^23^ of 150 min of physical activity per week, women were more likely to be inactive (43%) than men (26%). Women had worse quality of sleep compared with men.

Mean systolic blood pressure at age 22 years was 131.6 mmHg [standard deviation (SD) 12.3] for men and 116.9 mmHg (SD 11.0) for women, and mean diastolic pressure was 73.8 mmHg (SD 8.6) and 72.4 mmHg (SD 8.7), respectively. Forced expiratory volume in the first 1 s of forced vital capacity was higher in men [4.1 L (SD 0.6)] compared with women [3.0 L (SD 0.5)]. Men also showed better lung carbon monoxide diffusion than women.

## What are the main strengths and weaknesses? 

The main strengths of the study include its population basis and the wide range of data collected at different ages, allowing analyses of life course trajectories and of early determinants of health and human capital. The follow-up rate of 73.6% is higher than that reported by similar studies in low- and middle-income countries (LMIC).^9^ Another strength is the fact that the 1993 cohort is one of four cohorts (the others including all births in 1982, 2004 and 2015) carried out in the same city, with similar methods and measurements. This is a unique set of cohorts in LMICs that allows life course studies over a period of more than three decades, in a setting with rapid epidemiological and nutritional transitions.

In terms of weaknesses, the follow-up rate, as expected, was lower than that obtained in the previous follow-up at 18 years (81.4%), because as young people gain economic independence and move out of their parents’ home, it becomes more difficult to trace them. In the comparison between the original sample and those located at the age of 22 years, there were no statistical differences except for sex. At least 69% of subjects in any category of the baseline variables were traced (Table 1). Also, because visits take place with intervals of a few years, part of the information obtained may be affected by recall. Mortality information is based on official records, which may be incomplete (particularly for deaths that occurred outside Pelotas) or have poor quality of information on cause of death.

## Can I get hold of the data? Where can I find out more? 

All the documentation from the study is in Portuguese. Further details can be found on the website: [http://www.epidemio-ufpel.org.br/site/content/coorte_1993/index.php]. We welcome colleagues from other parts of Brazil or from abroad come to Pelotas to work with our team and analyse our datasets. We often host such researchers, as well as postgraduate students who may use our data for their dissertations. We also participate in a number of cross-cohort collaborations, including the COHORTS group^9^ and the RPS Consortium of Brazilian Cohorts, and have particularly strong collaboration with the ALSPAC cohort in the UK.^24^ For more information for proposed collaboration or to gain access to the 1993 Cohort data, potential partners should download the ‘Form for projects that do not involve data collection’ from this website or e-mail the corresponding author.

**Update in a nutshell**

- The 1993 Pelotas birth cohort includes all live births in the municipality of Pelotas, between 1 January and 31 December in the 1993 calendar year.
- The age of 22 years provides information on the beginning of adult life, enabling tstudy of the early determinants of markers of chronic diseases.
- The most recent evaluation was made between 2015 and 2016, and 3810 participants were analysed at a mean age of 22.6 years.
- The 22-year-old visit focused on four themes: (i) mental health; (ii) body composition and nutrition; (iii) precursors of complex chronic diseases; (iv) human capital.
- The 1993 Pelotas Birth Cohort has several external collaborators, including researchers living in middle- and high-income countries. For enquiries and new collaborative projects, visit the programme’s website at [http://www.epidemio-ufpel.org.br/site/content/], and contact [anamene.epi@gmail.com] or [hdgs.epi@gmail.com].

## Funding

This work was supported by the Science and Technology Department, Brazilian Ministry of Health, with resources transferred through the Brazilian National Council for Scientific and Technological Development (CNPq) (grant number 400943/2013–1). The study ‘Pelotas Birth Cohort, 1993’ was conducted by the Postgraduate Program in Epidemiology at Universidade Federal de Pelotas, with the collaboration of the Brazilian Public Health Association (ABRASCO). From 2004 to 2013, the Wellcome Trust supported the 1993 birth cohort study. The European Union, National Support Program for Centers of Excellence (PRONEX), the Brazilian National Research Council (CNPq), the Foundation for Research Support of the State of Rio Grande do Sul (FAPERGS) the Brazilian Ministry of Health supported previous phases of the study.
